# Simulation of Atrial Fibrosis Using Coupled Myocyte-Fibroblast Cellular and Human Atrial Models

**DOI:** 10.1155/2017/9463010

**Published:** 2017-12-26

**Authors:** Yuan Gao, Yinglan Gong, Ling Xia

**Affiliations:** Key Laboratory for Biomedical Engineering of Ministry of Education, Department of Biomedical Engineering, Zhejiang University, Hangzhou 310027, China

## Abstract

Atrial fibrosis is characterized by expansion of extracellular matrix and increase in the number of fibroblasts which has been associated with the development and maintenance of atrial arrhythmias. However, the mechanisms how the fibrosis contributes to atrial arrhythmia remain incompletely understood. In this study, we used a proposed fibroblast model coupled with the human atrial myocyte to investigate the effects of fibrosis on atrial excitability and repolarization at both cellular and macroscopic levels. The 12-lead electrocardiogram (ECG) was also simulated to explore the index of clinical diagnosis for fibrosis. The simulation results showed that the fibrosis can modify action potential morphology of human atrial myocyte, slow down wave propagation, and have rate adaptation, thus causing the atrial electrical heterogeneity. The fibrosis alone was sufficient to cause arrhythmia, induce reentry wave, and result in low amplitude and wide P waves at normal heart rate and significant prolonged and inverse P waves at high heart rate. All these symptoms aggravated when the level of fibrosis increased. Our simulations demonstrated that fibrosis is the substrate of atrial arrhythmia and thereby may be a potential target in the treatment of atrial arrhythmias.

## 1. Introduction

Fibrosis is a critical aspect of cardiac dysfunction following myocardial infarction, hypertension, heart failure, and severe arrhythmia [1, 2]. It is characterized by expansion of myocardial extracellular matrix and increase in the number of cardiac fibroblasts (Fbs). It increases with age and can lead to slow propagation and contribute to unidirectional block [3]. Apart from electrical remodeling and contractile dysfunction, atrial fibrosis has been shown to increase the susceptibility to atrial fibrillation (AF) and may serve as a critical substrate in the formation of the arrhythmia [4, 5]. A study about the small mouse atria has indicated that the alterations in atrial conduction produced by atrial interstitial fibrosis alone were sufficient to produce a substrate for AF [6].

But the mechanisms by how the fibrosis contributes to atrial arrhythmia remain incompletely understood. Cardiac fibroblast was the essential cell type in heart that is responsible for the homeostasis of the extracellular matrix. But it may transform to a myofibroblast phenotype and contribute to cardiac fibrosis when under impairment [7]. Many studies have revealed that myocytes and fibroblasts are functionally coupled by gap junctions when fibrosis occurred and may interact and modify impulse conduction via two-way electronic signal [8–10]. These results suggest a potential role for myocyte-fibroblast coupling in atrial arrhythmia.

Although there has been significant progress in the study of fibrosis over the past few decades, there still remain many unanswered questions. Investigation on human atrial myocardium with fibrosis is still very limited due to the difficulties in accessing experimental data. Therefore, alternative methods such as computer simulations are of great importance [11]. The main purpose of this study was to investigate the effects of fibrosis on atrial excitability and repolarization. To achieve this goal, we used a proposed fibroblast model [12] coupled with the Courtemanche et al. model [13] for atrial myocyte to stimulate the changes of action potential (AP) at the cellular level. At the macroscopic level, the AP models were incorporated into a human atrial model to simulate cardiac excitation conduction. Since the 12-lead ECG has been widely accepted as the main noninvasive diagnostic method of cardiac disease, we simulated the fibrosis ECG patterns to serve as an assistant method to study the underlying relationship between morphology of ECG and fibrosis atrial electrical activity.

## 2. Material and Methods

### 2.1. Myocyte-Fibroblast Electrophysiological Coupling

**Figure 1:**
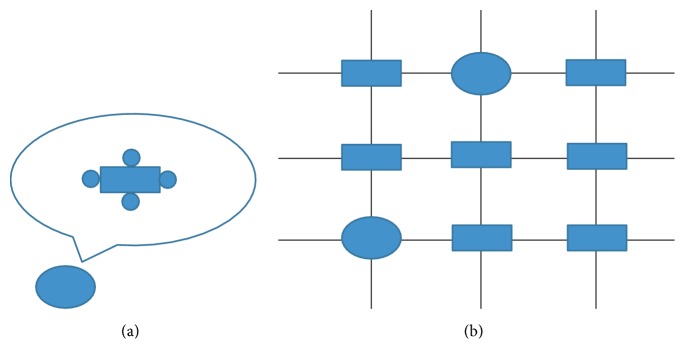
Illustration of myocyte/Fbs complex unit. The rectangle indicates a normal atrial myocyte, the ellipse indicates a myocyte/Fbs complex unit, and the circle around the myocyte indicates a fibroblast. (a) View of one myocyte/Fbs complex unit: a single human atrial myocyte is coupled a selected number of homogeneous fibroblasts via assigning an intercellular conductance (*G*gap) [14]. In this study, the number of fibroblasts was 1 to 6. (b) Schematic representation of 2D atrial tissue: myocyte/Fbs complex unit that replaces the normal myocyte random existing in the tissue indicates the fibrosis proliferous area [15].

In our simulation, the atrial myocyte was surrounded by a selected number of fibroblasts in normal tissue, which was regarded as one myocyte/Fbs complex unit. The paradigm is illustrated in Figure 1.

The electrophysiological model of atrial myocyte is based on the Courtemanche et al. [13], which can accurately represent the atrial AP dynamics. Using specific formulations of Na^+^, K^+^, and Ca^2+^ currents based on the experimental data recorded from human atrial myocytes, along with representations of pump, exchange, and background currents, the model is computationally efficient and correctly replicates physiologically repolarization process.

The atrial fibroblast model was based on the active 1 model described by Maleckar et al. [12]. The model contains four active membrane ionic currents: time and voltage dependent K^+^ current, inward-rectifying K^+^ current, Na^+^-K^+^ pump current, and background Na^+^ current. This model originates from a previous mathematical formulation of MacCannell et al. [14] and is modified based on the experimental data to well represent human atrial fibroblast properties. The membrane potential of the coupling model is governed by the following equation:

(1) dVmyodt=−1CmyoImyoVmyo,t+Istim+∑i=1:nIgap,dVFbdt=−1CFbIFbVFb,t−Igap,Igap=GgapVmyo−VFb,

where *V*_myo_ and *V*_Fb_ represent the membrane potential of the atrial myocyte and fibroblast, respectively, *C*_myo_ and *C*_Fb_ are the membrane capacitance of the myocyte and fibroblast, respectively, *I*_myo_ is the net membrane current of the myocyte, and *I*_Fb_ is the net membrane current of fibroblast. *I*_stim_ is the stimulus current applied to myocyte membrane, *I*_gap_ is the current that flows through the gap junction between the myocyte and each fibroblast, *n* is the total number of fibroblasts, and *G*_gap_ represents the gap junction conductance. According to the experimental reports, *C*_Fb_ ranges from 6.3 to 75 pF [16]; *G*_gap_ between a myocyte and a fibroblast ranges from 0.3 to 8 nS in cultured cells [17]. In this study, we used *C*_myo_ = 100 pF, *C*_Fb_ = 6.3 pF, and *G*_gap_ = 3 nS.

### 2.2. Model of 3D Atria

The atrial specimen was obtained from a healthy male adult in Zhujiang Hospital, Southern Medical University, China. The use of the heart for research purpose was approved by the local Ethics Committee of the Southern Medical University. The National Rules and Regulations on Heart research were strictly followed. The specimen was scanned by spiral computer tomography (Philips/Brilliance 64) with a resolution of 512 pixels by 512 pixels and a spatial resolution of 0.3574 × 0.3574 × 0.33 mm. Details of the model were described in our previous study [18–20].

In the model, the conduction system included sinoatrial node (SAN), Bachmann's bundle (BB), crista terminalis (CT), pectinate muscles (PM), slow pathway (SP), and fast pathway (FP). In order to simulate the anisotropy, the fiber orientation of atria was contained. During the propagation, each myocardial unit has specific electrophysiological parameters associated with the action potential of the cell unit and conduction velocity.

The propagation of action potential was based on the monodomain model [21]:

(2) $$\begin{array}{c} \frac{{\partial V}_{m}}{\partial t}=\frac{1}{C_{m}}\left(\;\frac{1}{A_{m}}\left(\;\frac{\lambda }{1+\lambda }\nabla \cdot \left(\;\sigma_{i}\nabla V_{m}\;\right)-I_{ion}+I_{app}\;\right)\;\right), \end{array}$$

where *V*_*m*_ is transmembrane voltage, *C*_*m*_ is the membrane capacitance, *A*_*m*_ is surface-to-volume ratio, and we used *A*_*m*_ = 1000 cm^−1^ and *C*_*m*_ = 1 *μ*F/cm^2^ [22]. *λ* is the ratio of conductivity extracellular to intracellular, *σ*_*i*_ is cellular conductivity, and conductivity values were based on experimental data studied by Roth [23]. The transversal conductivity was set to 0.15 S/m for myocyte and CT, 0.11 S/m for PM and BB, and 0.05 S/m for SAN. The ratio for cross-axis to long-axis conductivity was set to 1 : 9. *λ* was set to 1.0. *I*_ion_ is the sum of ionic currents, and *I*_app_ is the sum of applied stimulus currents.

The equation was solved numerically using explicit Euler method based on parallel computational techniques. The simulations were performed on a cluster of networked Dawning TC4000L system. The hardware architecture is symmetric multiprocessor shared memory that contains one management node and ten computation nodes. Each computation node consisted of two Intel Xeon 5335 processors and 4 GB of shared memory. The total theoretical computing capacity can be up to 184 Gflops. We used MPI to implement the communication between nodes.

The torso model in our study was taken from the virtual male subject of the United States. The body surface potentials generated by the cardiac sources satisfy the Poisson equation with Newman boundary conditions:

(3) ∇·σ∇Φ=−∇·Js,in  Ω,σ∇Φ·n=0,on  SB,

where *σ* is the tissue dependent conductivity tensor, Φ is the quasi static potential, **J**_*s*_ is the density of the equivalent dipole sources, **n** is the normal vector, *S*_*B*_ is the body surface, which encloses the volume conductor *Ω*.

Using the Green second identity,

(4) $$\begin{array}{c} \int_{S}\left(\;A\nabla B-B\nabla A\;\right)\cdot n\;dS=\int_{V}\left(\;A\nabla^{2}B-B\nabla^{2}A\;\right)d\Omega \end{array}$$

with

(5) A=1R,B=σΦ.

The differential equation for Φ as (3) can be solved as the following integral equation:

(6) Φr=14πσ∫ΩhJs·∇1R dV+∑l=1mσl+−σl−∫SlΦr∇1R dS,

where **R** = **r** − **r**_*s*_ is the vector between the field point **r** and source point **r**_*s*_; *Ω*_*h*_ is the heart area, *S*_*l*_ is the conductivity junction surface, and its inside and outside conductivities are *σ*_*l*_^−^ and *σ*_*l*_^+^, respectively. Further details of the model can be found in our previous studies [24, 25].

The 12-leads ECGs are calculated as [26]

(7) I=VLA−VRA,II=VLL−VRA,III=VLL−VLA,aVR=−12I+II,aVL=I−12II,aVF=II−12I,Vi=VPi−VLA+VRA+VLL3,

where *V*_LA_ is left arm surface potential, *V*_RA_ is right arm surface potential, *V*_LL_ is left leg surface potential, *V*_*i*_ is each precordial lead (*i* = 1,2,…, 6), and *V*_*Pi*_ is each precordial surface potential.

## 3. Results

### 3.1. Action Potential of Atrial Myocyte Coupling Fibroblasts

In our study, we first simulated the APs of one human atrial myocyte coupled different number of fibroblasts with a *G*_gap_ of 3 nS at stimulation frequencies between 1 and 3 Hz. More than 5 to 10 stimuli were applied to achieve steady state conditions.

**Figure 2:**
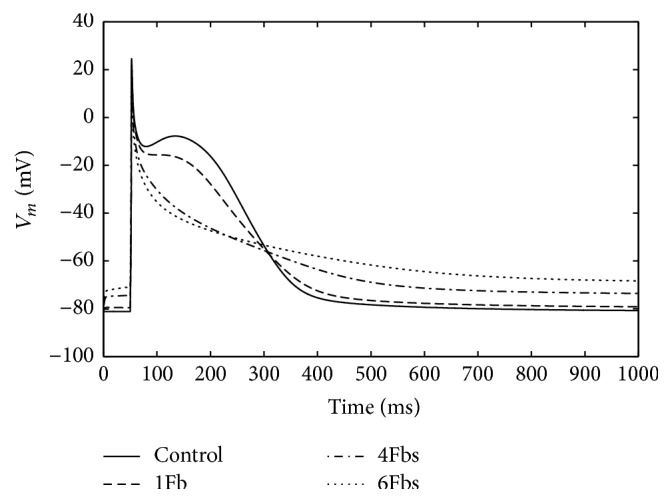
Illustration of changes in the waveform of atrial myocyte action potential when coupling to Fbs with a *G*_gap_ of 3 nS for 1 Hz pacing.

Figure 2 shows the APs of atrial myocyte when coupled with 1, 4, and 6 fibroblasts, respectively, in comparison with the noncoupled control for pacing at 1 Hz. In Table 1, we compare typical characteristics of action potentials for the coupled myocyte. By comparison, we can see that, with more coupled fibroblasts, the maximum overshoot potential is decreased and resting potential is increased. When the number of fibroblasts increased, the membrane potential during the plateau was less depolarized, and the AP duration to 60% repolarization (APD_60_) decreased quickly. But the 90% repolarization (APD_90_) was prolonged with the increasing of fibroblasts.

**Figure 3:**
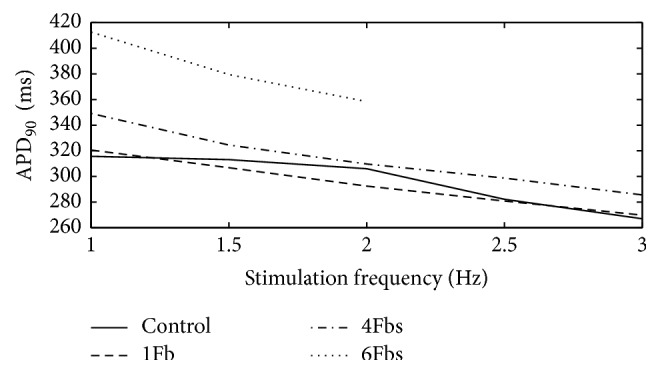
APD_90_ of one atrial myocyte coupled Fbs at different stimulation frequencies.

Figure 3 shows the APD_90_ of atrial myocyte coupled fibroblasts at stimulation frequencies between 1 and 3 Hz. Each of the cells displayed marked frequency dependent adaptation of the APD with a shortening at higher stimulation rates. The APD of noncoupled myocyte changed smoothly at low rates and sharply at high rates. However, the APD of coupled myocyte changed linearly with the rate increased. Remarkably, when coupled with 6 fibroblasts, the myocyte failed to achieve steady state condition at high frequency.

### 3.2. Simulation of 3D Atrial Exciting Sequence Map and 12-Lead ECG

Although the ratio has not been conclusive, fibrosis is characterized by the increase in the number of fibroblasts [27]. In this study, we simulated one atrial myocyte coupled 4 fibroblasts as a myocyte/Fbs complex unit. Then such a unit was used to randomly replace one normal myocyte in atria to simulate fibrosis. 10% and 70% of total atrial myocytes were replaced by the units to simulate gentle and severe fibrosis.

**Figure 4:**
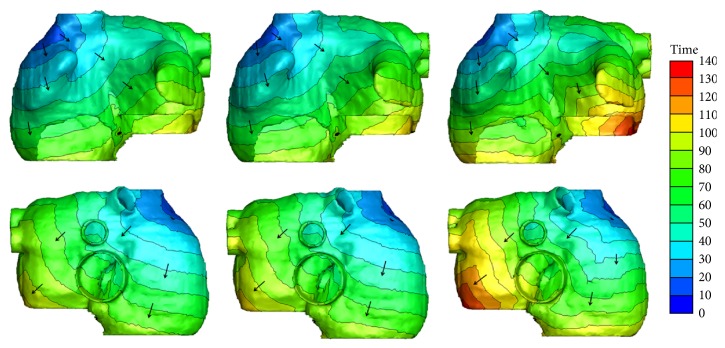
Simulated activation sequences at 1 Hz pacing. First column: normal atria; second column: atria with 10% fibrosis; third column: atria with 70% fibrosis. The first row is the anterior view of the atria; the second row is the posterior view of atria. The arrows indicate the direction of the wave propagation. The color bar on the right-hand side indicates the propagation time with the units in milliseconds.

Figure 4 shows the exciting sequence map of atria with 10% and 70% fibrosis at 1 Hz pacing (i.e., the sinus rhythm was 60 beats per minute). For comparison, the exciting sequence map of normal atria was also presented. The first column of Figure 4 shows the typical normal atrial exciting sequence maps. The depolarization duration of the right atrium was 86 ms. The last part to be activated was at the site below the inferior vena cava. The total depolarization duration of the atria was 102 ms at the place of posterior left atrial wall. The second column shows the exciting sequence maps of the atria with 10% fibrosis. The right atrial depolarization duration was 90 ms and the total depolarization duration was 106 ms. The last activated areas were nearly the same as the normal case. The third column was the exciting sequence maps of the atria with 70% fibrosis. The last place to be activated in the right atrium was the right lateral wall at the time of 104 ms. The total depolarization duration of the atria was 133 ms nearly at the same place as the normal case.

**Figure 5:**
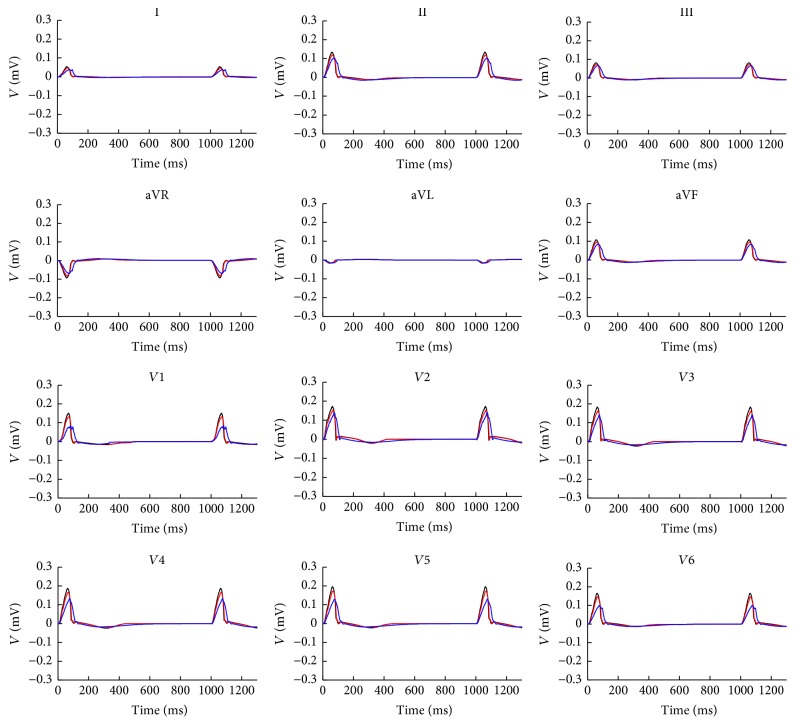
12-lead ECG at 1 Hz in two cycles. The black lines are the normal case, red lines are atria with 10% fibrosis, and blue lines are the atria with 70% fibrosis.

Figure 5 shows the 12-lead ECG of atrium with 10% and 70% fibrosis in comparison with normal atria at 1 Hz pacing in two cardiac cycles. Because the fibrosis decreased the cellular maximum overshoot potential and reduced the atrial electrical excitation, this led to the smaller amplitude of P wave on each lead. As the fibrosis prolonged the atrial excitation time, the P wave duration was extended. The phenomenon has an increasing trend with the fibrosis degree growing. Since the time of atrial repolarization was sufficient, ECG patterns in the second cycle did not significantly change.

**Figure 6:**
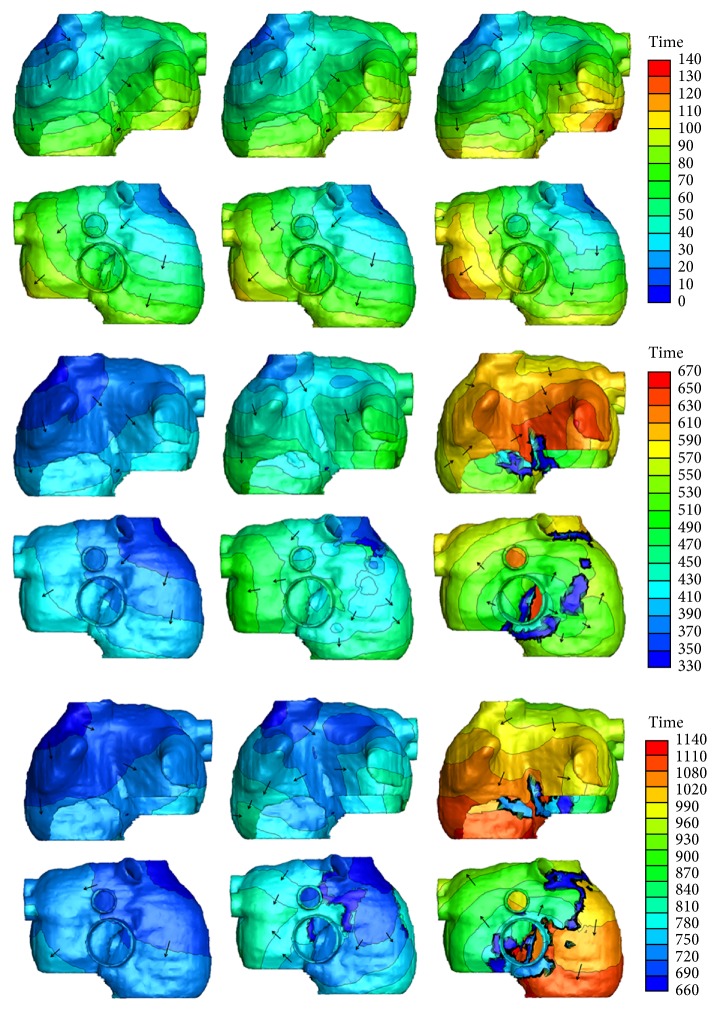
Simulated activation sequences at 3 Hz pacing. First column: normal atria; second column: atria with 10% fibrosis; third column: atria with 70% fibrosis. First two rows: anterior and posterior view of first cardiac cycle. Since the total activation duration is less than 133 ms, time window of the beginning 140 ms was given. Middle two rows: anterior and posterior view of second cardiac cycle. Last two rows: anterior and posterior view of third cardiac cycle. The arrows indicate the direction of the wave propagation. The color bars on the right-hand side indicate the propagation time with the units in milliseconds.

**Figure 7:**
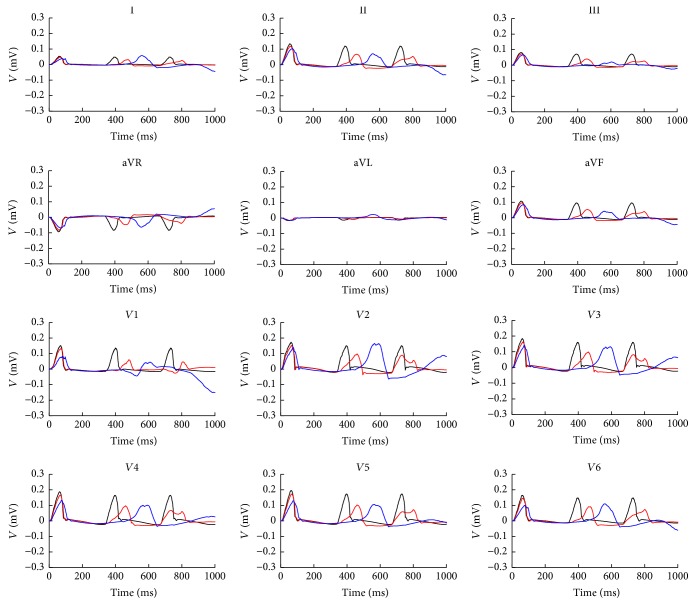
12-lead ECG at 3 Hz in three cycles. The black lines are the normal case, red lines are atria with 10% fibrosis, and blue lines are the atria with 70% fibrosis.

Figure 6 shows the exciting sequence map of atria with 10% and 70% fibrosis at 3 Hz pacing in three cardiac cycles. The first cycle of the excitation patterns was the same as Figure 4. The conduction pattern of atria with 10% fibrosis within the second cycle was slightly different. Since the repolarization of atrial myocytes near the proximal ends of conductive bundles was not sufficient, the excitation potential was far below the normal level. This led to the slow excitation propagation in the atria and slightly changed the exciting sequence. So the early part of P wave was gentle and an obvious wide P wave was visible on each lead (see Figure 7). The conduction pattern of atria with 10% fibrosis in the third cycle was more unusual. A quick exciting wave was initiated at the right posterior wall propagating around inferior vena cava from right atrium to the left atrium and merged with the planner wave propagated from the left atrial roof. It also propagated retrograde to the right lateral wall and merged with the wave propagated from the right anterior wall. This led to the notches of P waves appearing on most leads and inverse P wave on lead I.

As the fibrosis level increased to 70%, the myocytes near the sinoatrial node failed to be activated in the second cycle. The first exciting area was the right posterior wall below the inferior vena cava, initiating the reentry wave meandered in both the left atrium and the right atrium. The total activating time persisted nearly the whole cardiac cycle and merged with the next cycle. So the prolonged and inverse P waves were more significant in the ECG patterns.

## 4. Discussions

In this study, based on a coupled myocyte-fibroblast model, we investigated the effects of fibrosis on human atria at both cellular and macroscopic level. At the cellular level, the AP morphology of the atrial myocyte is modified remarkably during depolarization and repolarization. With the number of coupled fibroblasts increasing, the resting membrane potential elevated and leads to weakened excitability along with the maximum overshoot potential decreased. Curtailment of 60% repolarization was seen and plateau of the AP disappeared depending on the increased number of coupled fibroblasts. However, the APD_90_ was prolonged with the increasing of fibroblasts. The atrial myocyte also displayed marked frequency dependent on the APD with a shortening at higher stimulation rate.

At the macroscopic level, the fibrosis changed the exciting wave pattern in the atria. When the sinus rhythm was normal, the velocity of wave propagation slowed down with the increased level of fibrosis, leading to the total duration of atrial depolarization and repolarization being prolonged. This resulted in the smaller amplitude and wider P wave on each lead. Since the time of atrial repolarization was sufficient, the excitation and ECG patterns at each cardiac cycle did not have obvious change. With the heart rate increasing, the electrical anomalous conduction in the atria was more obvious. Local electrical conduction block and reentry waves were initiated and meandered in both the right atrium and the left atrium. Notches of P waves appeared on most leads; significant prolonged and inverse P waves were observed. The reduced amplitude and prolonged duration of P waves have been observed in previous ECG recordings of animal and human with atrial fibrosis [28–31]. But there was no report about inverse P wave in patient with atrial fibrosis. However, this morphology has been shown to be common in patients with AF [32–35]. Notably, other morphologies of P waves in our simulation (e.g., wide duration and notches) also can be seen in these patients. This suggested the possibility that patients with atrial fibrosis can show similar ECG patterns characterized by AF.

Since atrial fibrosis is associated with a variety of cardiomyopathies and one of the main factors determining the relapse after therapies, the evaluating of existence and level of atrial fibrosis in atria has increasing importance for therapy strategies [5]. Nowadays late gadolinium-enhanced MRI (LGE-MRI) is the mainstream technique for detecting fibrosis [36]. But the image quality of LGE-MRI scans is frequently poor due to various reasons (e.g., residual respiratory motion, heart rate variability, low resolution limited by the thinness of atrial wall, and confounded enhancement from surrounding heart substructures). These caused a large number of false positives in the atrial fibrosis delineation. ECG may serve as a clinical method to guide the LGE-MRI evaluation. This could have implications for treatment as well as future clinical trials. Our simulations provide a promising starting point for assessing patients with atrial fibrosis by ECG, and this could provide some references for clinical diagnosis.

It should be point out that there are still some limitations in this study. In our simulation, the atrial fibrosis model was constructed by uniformly random distributed fibrosis but not patient-derived. At the macroscopic level, we only considered the percentage increasing to represent the enhancement of fibrotic level, and the different number of coupled fibroblasts was not taken into account. Finally, the model used in this study was a static heart model with electrophysiological properties, but the mechanical functions have not been considered. Cardiac motion should be considered in future studies to further improve the simulation accuracy.

## 5. Conclusions

In this study, a coupled myocyte-fibroblast model has been used to investigate the effects of atrial fibrosis at cellular and human atrial levels. The 12-lead ECG was also simulated to explore the index of clinical diagnosis of fibrosis. The results show that the fibrosis can modify AP morphology of human atrial myocyte, slow down wave propagation, and have rate adaptation. These caused the atrial electrical heterogeneity. The fibrosis alone was sufficient to cause arrhythmia, induce reentry wave, and result in low amplitude and wide P waves at normal heart rate and significant prolonged and inverse P waves at high heart rate. These symptoms will aggravate with the level of fibrosis increased. Our simulations demonstrated that fibrosis is the substrate of atrial arrhythmia and thereby may be a potential target in treatment of atrial arrhythmia. The understanding of the ECG changes could provide some references for clinical diagnosis.

## Figures and Tables

**Table 1:** Characteristics for an atrial myocyte coupled Fbs with a *G*_gap_ of 3 nS for 1 Hz pacing.

#FB	*V*_rest_ (mV)	*V*_max_ (mV)	APD60 (ms)	APD90 (ms)
0	−81.2	24.4	230.8	315.7
1	−79.6	23.2	210.6	320.6
4	−74.1	18.1	114.0	349.1
6	−69.2	6.5	71.6	412.6

#FB indicates the number of fibroblasts coupled myocyte, *V*_rest_ indicates the resting potential, and *V*_max_ is the maximum overshoot value.
